# Molecular Epidemiology of Human Metapneumovirus Infections in Children from San Luis Potosí-Mexico

**DOI:** 10.3390/v17101338

**Published:** 2025-10-02

**Authors:** Nadia Martínez-Marrero, Juan Carlos Muñoz-Escalante, Jan Michell Yerena-Rivera, Luis Rubén Jaime-Rocha, José J. Leija-Martínez, Ana María González-Ortiz, Daniel E. Noyola

**Affiliations:** 1 Infectious Diseases Laboratory, Centro de Investigación en Ciencias de la Salud y Biomedicina, Universidad Autónoma de San Luis Potosí, Av. Sierra Leona 550, Colonia Lomas de San Luis, San Luis Potosí 78210, SLP, Mexico; nadiamar24@gmail.com (N.M.-M.); carlos.escalante@uaslp.mx (J.C.M.-E.); janyerena3@gmail.com (J.M.Y.-R.); jesus.leija@uaslp.mx (J.J.L.-M.); 2 Microbiology Department, Facultad de Medicina, Universidad Autónoma de San Luis Potosí, Av. Venustiano Carranza 2405, Colonia Lomas de los Filtros, San Luis Potosí 78210, SLP, Mexico; 3 Hospital de la Niñez y la Mujer “Dr. Alberto López Hermosa”, Blvd. Antonio Rocha Cordero 2510, San Luis Potosí 78364, SLP, Mexico; anagon71@yahoo.com.mx

**Keywords:** human metapneumovirus, molecular epidemiology, acute respiratory infections, Pneumovirus, glycosylation

## Abstract

Lower respiratory infections are a leading cause of death in children under five years. Human metapneumovirus (HMPV) is an underestimated causal agent of these infections. In this study, the molecular epidemiology of HMPV associated with respiratory infections in Mexican children between August 2023 and August 2024 was determined. Sequences were also analyzed for predicted N- and O-linked glycosylation sites. Overall, 34 sequences from infants with respiratory infections were obtained; 32 were assigned to the A2b2 genotype, one to A2b1, and one to B2. All but one of the A2b2 sequences carried the 111-nucleotide duplication of the G gene; the remaining sequence carried the 180-nucleotide duplication. The samples assigned to the A2b1 and B2 genotypes did not have a duplication. The HMPV-A phylogeny did not show a clustering of Mexican sequences as a single monophyletic group. Four N-linked glycosylation sites were predicted in the HMPV-A sequences and three in the B sequence. The number of O-linked glycosylation sites predicted in HMPV-A ranged from 61 to 77 and were 61 in the HMPV-B sequence. This first description of HMPV genotypes and the diverse array of G protein N- and O-linked glycosylation patterns found in a Mexican pediatric population in the post-pandemic period contributes to the understanding of the global spread of HMPV.

## 1. Introduction

Human metapneumovirus (HMPV) is a significant and underestimated causal agent of acute respiratory infections in children under five years. HMPV accounts for a significant proportion of lower respiratory tract infections (LRTI) in infants and young children [1,2]. Generally, infants and young children diagnosed with HMPV infection present symptoms similar to those caused by other respiratory viruses, including rhinorrhea, fever, and cough [3]. However, in patients requiring hospitalization, the most common diagnoses are bronchiolitis and pneumonia [4]. In addition, HMPV infections have occasionally been associated with central nervous system manifestations ranging from self-limiting disorders, such as febrile seizures, to severe presentations, such as encephalitis [5]. Besides age, risk factors associated with severe HMPV infection include preterm birth, underlying chronic pulmonary, heart, or neurologic disorders, and immunosuppression [6].

HMPV has been reported as a cause of respiratory infections on all continents, with a seasonal epidemiology that was significantly altered during the COVID-19 pandemic [7,8,9,10]. Non-pharmacological interventions instituted to reduce the spread of SARS-CoV resulted in a reduced number of HMPV infections during 2020. The reduced exposure to this pathogen during that time led to a reduction in protective immunity in the community [10]. Once social distancing measures to prevent the spread of SARS-CoV-2 were lifted, sudden off-season epidemics of HMPV infections occurred, surpassing pre-pandemic levels [10,11,12].

HPMV is an enveloped virus and a member of the *Pneumoviridae* family [13,14]. It has a negative-sense, single-stranded RNA genome, and the gene encoding for the attachment glycoprotein (G gene) shows the highest sequence variability [14]. The G glycoprotein, a predicted mucin-like type II transmembrane protein, mediates viral attachment to cell receptors and could also participate in the suppression of host innate immune responses [15]. Van den Hoogen et al. [16] defined two HMPV groups (A and B) based on sequence diversity between G genes and differences in virus neutralization titers. Subsequently, these groups were divided into subgroups or genotypes: A1, A2, B1, and B2 [17]. The evolution of HMPV has led to further diversity. Two distinct clades, termed A2a and A2b, have been identified within genotype A2 [18]. Furthermore, molecular evolutionary analyses indicate that two lineages, A2b1 and A2b2, diverged from clade A2b approximately a decade after genotype A2 split into A2a and A2b [19]. Clade A2b1 includes older HMPV sequences and continues to be referred to as A2b by many authors and more recently as A2.2.1 by others. Clade A2b2 encompasses the most recent HMPV isolates, including viruses with partial duplications of the G gene (111 or 180 nucleotides (nt) in length) [20,21]. Clade A2b2 has also been referred to as A2c, Novel A2 sublineage, Unique A2 sublineage, Unique A2b sublineage, and A2.2.2 [19,22,23,24].

The epidemiological changes of HMPV infections observed during and after the pandemic, as well as the emergence of novel variants of this virus, such as those presenting partial duplications of the G gene, highlight the importance of carrying out epidemiological studies in all world regions. In this regard, few epidemiological studies on HMPV have been made in Mexico [25,26] and only one study, carried out 20 years ago, has previously reported the HMPV genotype distribution [25]. As such, the present study constitutes the first approximation of the current molecular epidemiology of HMPV in Mexico. The aim of this work was to determine the HMPV genotypes associated with respiratory infections in a group of Mexican children between August 2023 and August 2024 to contribute to the understanding of global HMPV evolution and generate a baseline to compare with future molecular epidemiology analyses in Mexico. In addition, the study analyzes the predicted N- and O-linked glycosylation sites in the G protein as an assessment of the potential relevance of the genotypic features of strains currently circulating in diverse world regions.

## 2. Materials and Methods

### 2.1. Study Population and Samples

The study population included 390 children with LRTI admitted to the Hospital del Niño y la Mujer “Dr. Alberto López Hermosa” in San Luis Potosí (SLP) State, Mexico, between August 2023 and August 2024. Prior to study participation and sample collection, the parents of children were invited to participate and written informed consent was obtained. After collection, samples were transported to the laboratory under refrigeration conditions. The study was approved by the Research and Ethics Committee of the Ministry of Health of the Government of the State of San Luis Potosí (SLP/04-2023; date of approval: 3 August 2023). The clinical characteristics and viral agents detected in the study population have been reported previously [27]. The present work focuses on the molecular epidemiology of HMPV through a detailed analysis of the viral G gene, since no molecular studies of this virus have been carried out in the last 20 years in Mexico and only three HMPV sequences from infections identified in Mexico were available at GenBank.

### 2.2. RNA Isolation, Reverse Transcription, and qPCR for HMPV Detection

RNA was isolated from 140 μL of respiratory samples using QIAamp Viral RNA Mini Kit (QIAGEN, Hilden, Germany) and suspended in 60 μL of elution buffer in accordance with the manufacturer’s instructions. Reverse transcription reactions were carried out with a RevertAid H Minus First Strand cDNA Synthesis Kit (Thermo Fisher Scientific Inc., Waltham, MA, USA). Random primers and 250 ng of RNA template were used. The total cDNA was analyzed for the presence of HMPV. The final reaction volume was 12.5 μL containing 1X of Maxima SYBR Green/ROX qPCR Master Mix (Thermo Fisher Scientific Inc., Waltham, MA, USA) and 0.3 mM of specific primers NIID-N1-F and NIID-N1-R (Table S1, [28]) for the amplification of an N-gene fragment, using the CFX Opus 96 Real-Time PCR System for detection. Cycling conditions were as follows: An initial denaturation at 95 °C for 10 min followed by 40 cycles of denaturation at 95 °C for 15 s, annealing at 54.5 °C for 30 s, and an extension at 72 °C for 30 s. The cut-off cycle threshold (Ct) value used to consider a sample as positive was 38.

### 2.3. HMPV-A and HMPV-B Genotyping

Genotyping was carried out in HMPV samples with high viral loads. Samples with Ct values <30 were selected for genotype analysis since samples with low viral loads (i.e., Ct values of 30 or more) are notably less likely to yield sequences for analysis. Genotyping was performed by sequencing PCR products obtained with sets of primers for specific amplification of HMPV-A and HMPV-B G gene fragments (Supplementary Table S1, [16,20]). Amplification reactions were done in 25 μL of final volume with 1X DreamTaqTM Buffer + 2 mM de MgCl_2_ (Thermo Fisher Scientific Inc., Waltham, MA, USA), 0.2 mM of dNTPs mix, 0.3 µM of primers, and 1.25 U of DreamTaqTM DNA Polymerase (Thermo Fisher Scientific Inc., Waltham, MA, USA). Considering the detection qPCR Ct values (16–30), 1–2.5 μL of cDNA were used as template. The thermocycling conditions for PCR were 95 °C for 3 min, followed by 40 cycles of 30 s at 95 °C, 30 s at annealing temperature (Supplementary Table S1) and 1 min at 72 °C, followed by a final step at 72 °C for 5 min. All amplifications were made in an Applied Biosystems™ Veriti thermal cycler.

Oligonucleotide pairs for PCR generate amplicons of approximately 1000 bp for both HMPV-A and HMPV-B, which were observed using 1% agarose gels. Five to ten microliters of amplification products were purified with ExoSAP-IT Express (Applied Biosystems, Foster City, CA, USA) as suggested by the manufacturer and submitted for Sanger Sequencing using the BigDye Terminator Sequencing Kit v1.1 and an ABI 3500 Genetic Analyzer (both Applied Biosystems, Foster City, CA, USA), in LANBAMA-IPICYT-SLP. Sequences were manually edited with BioEdit v7.7.1 [29].

### 2.4. Global HMPV Sequences

A total of 5592 HMPV sequences were downloaded from GenBank on 15 January 2025, using taxonomy (taxid 162145) and sequence length filters (>500 nucleotide length). The HMPV sequences were aligned with MAFFT version 7.450 (https://mafft.cbrc.jp/alignment/server/index.html, accessed on 29 January 2025) using Reference Sequences to separate HMPV-A and HMPV-B sequences for subsequent analysis. Inspections and corrections of alignments were manually done with BioEdit v.7.7.1 [29]. Alignments were trimmed to limit the analysis to the G gene using HMPV-A (NC_039199) and HMPV-B (KC562244) as reference sequences. To avoid redundancy during phylogenetic analysis, identical sequences were removed from the final alignment using ElimDupes (https://www.hiv.lanl.gov/content/sequence/elimdupesv2/elimdupes.html, accessed on 29 January 2025). After excluding sequences that did not correspond to the G gene or contained more than 5% ambiguous nucleotides, 1840 HMPV-A and 1581 HMPV-B sequences met the criteria for phylogenetic analysis. Additionally, 33 HMPV-A sequences and one HMPV-B sequence obtained in this study were included in the phylogenetic analysis. Sample information (including collection date and geographic location) from sequences comprising the final HMPV-A and HMPV-B datasets was registered.

### 2.5. Recombination Analysis

To maximize the accuracy of the phylogenetic analysis, the dataset was analyzed to assess if recombination signals were present in any sequence using RDP5 v5.64 [30]. Possible recombination events were evaluated using the RDP, GENECONV, Chimaera, Max-Chi, SiScan, and 3Seq algorithms. True recombination events were defined as those detected by at least three of the six different algorithms used.

### 2.6. Phylogenetic Analysis

A fragment of the G gene sequences (860 bp for HMPV-A, and 730 bp for HMPV-B) encompassing nearly the entire gene, was used for phylogenetic analysis and lineage assignment. Phylogenetic inference for the HMPV-A and HMPV-B sequence datasets was performed with IQ-TREE v2.2.2.6 (http://www.iqtree.org, accessed on 30 January 2025) under the maximum likelihood method, applying the best-fitting substitution model as determined by the ModelFinder tool (for both the HMPV-A and HMPV-B datasets) integrated in IQ-TREE v2.2.2.6. Trees were visualized using FigTree software v.1.4.4 (http://tree.bio.ed.ac.uk/software/figtree/, accessed on 30 January 2025). Branch support was assessed using the SH-aLRT and UFBoot2 methods, with 1000 and 10,000 replicates, respectively. Monophyletic clades were considered statistically supported when the SH-aLRT value was >80% and the UFBoot2 value was >90%.

### 2.7. Time-Scaled Phylogenetic Analysis and Evolutionary Rate Estimation

To provide a temporal framework to the phylogenetic inferences, maximum likelihood (ML) trees generated with IQ-TREE were subsequently calibrated in time using TreeTime v.0.11.4.26 (https://github.com/neherlab/treetime, accessed on 30 January 2025). Sample collection dates were included as metadata for each sequence to scale branch lengths according to time, obtaining time-scaled phylogenies. Molecular evolutionary rates (substitution per site per year) were also estimated from the dataset.

### 2.8. Lineage Assignment

The phylogenetic trees were rooted against the A1 (for the HMPV-A dataset) or B1 (for the HMPV-B dataset) clades. Lineages were assigned to every sequence based on clustering with previously published reference sequences (Supplementary Table S2) within well-supported clades (bootstrap values > 75%). Lineage assignment information was added to the geo-temporal information data for subsequent analysis.

### 2.9. Glycosylation Site Analysis

Deduced amino acid sequences were obtained by BioEdit 7.7.1 [29]. A fragment of the G protein transmembrane region (amino acid positions 30–51) was analyzed in 29/34 sequences, and the complete ectodomain was studied in all 34 sequences. To predict N-linked and O-linked glycosylation sites in the aa sequences, the bioinformatics tools NetNGlyc v1 (sequon Asn-Xaa-Ser/Thr, *p* ≥ 0.5 or 0.75) and NetOGlyc v4 (*p* ≥ 0.5) (https://services.healthtech.dtu.dk/services/NetOGlyc-4.0/, accessed on 30 January 2025) were used.

## 3. Results

### 3.1. HMPV Detection in Infants with LRTI

HMPV was detected in 68/390 (17.4%) of the samples. In 37/68 (54.4%) of the HMPV positive samples other viruses were also detected: 18 (26.5%) HMPV + human respiratory syncytial virus (HRSV); 5 (7.4%) HMPV + SARS-CoV-2; 5 (7.4%) HMPV + influenza; 7 (10.3%) HMPV + HRSV + SARS-CoV-2; 1 (1.5%) HMPV + HRSV + influenza; and 1 (1.5%) HMPV + SARS-CoV-2 + influenza [27]. HMPV was detected as the only pathogen in 31/68 of the samples. The Cts of the positive HMPV samples ranged between 15.3 and 37.8, and 44 samples with a Ct < 30 were considered suitable for genotype analysis.

### 3.2. HMPV-A and HMPV-B Genotyping

Positive amplification for sequencing was obtained with HMPV-A specific primers in 33 samples, and one sample was positive using HMPV-B specific primers. As such, a G gene sequence fragment was obtained from 34 of the 44 (77.3%) positive samples with Ct < 30. Overall, HMPV samples from 50% (34 of 68) of the children with infections caused by this virus were characterized. To assess whether there were significant differences between children in whom sequences were obtained and those from whom no sequences were obtained, we compared the demographic and clinical characteristics between the children in these two groups. There were no significant differences in any characteristic between the children from whom sequences were obtained and those from whom no sequences were obtained, as shown in Supplementary Table S3, suggesting that the genotype distribution observed is representative of the complete study group. The sequences generated in the present study are available at GenBank (GenBank accession numbers: PV950564-PV950597).

### 3.3. Recombination Analysis

The presence of potential recombination signals was evaluated with RDP5 v.5.64 using the RDP, GENECONV, Chimaera, Max-Chi, SiScan, and 3Seq algorithms. In this analysis, we included the dataset which comprised 1840 HMPV-A and 1581 HMPV-B sequences from public repositories and the 34 sequences (33 HMPV-A sequences and one HMPV-B sequence) obtained in this study. No sequence from San Luis Potosí or from the global dataset fulfilled the criteria to be considered a true recombination event (detection by at least three independent algorithms).

### 3.4. Phylogenetic Analysis

**Figure 1:**
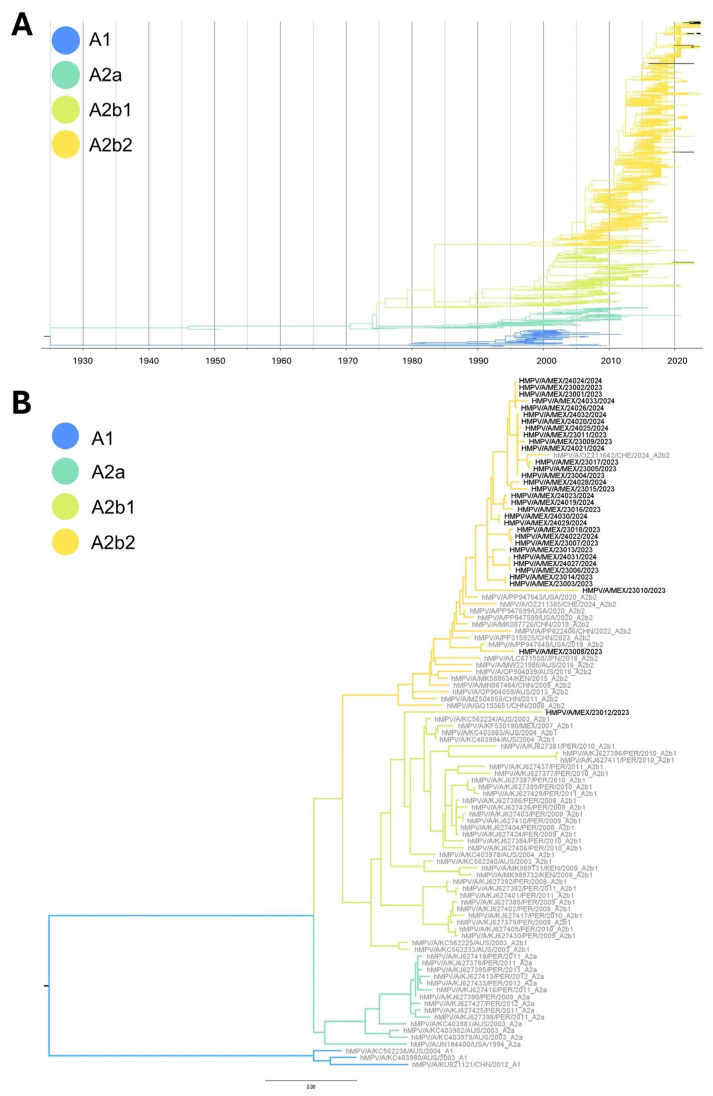
(**A**) Time-scaled maximum likelihood phylogenetic tree based on the global available G gene sequences of human metapneumovirus A (hMPV-A). Branches are colored according to their lineage assignment (A1, A2a, A2b1, A2b2), while sequences obtained and characterized in this study are shown in black. (**B**) Maximum likelihood phylogenetic tree including only hMPV-A sequences obtained in this study (black-labeled taxa) and representative reference sequences (gray-labeled taxa), highlighting the phylogenetic placement of the newly characterized viruses within hMPV-A lineages.

**Figure 2:**
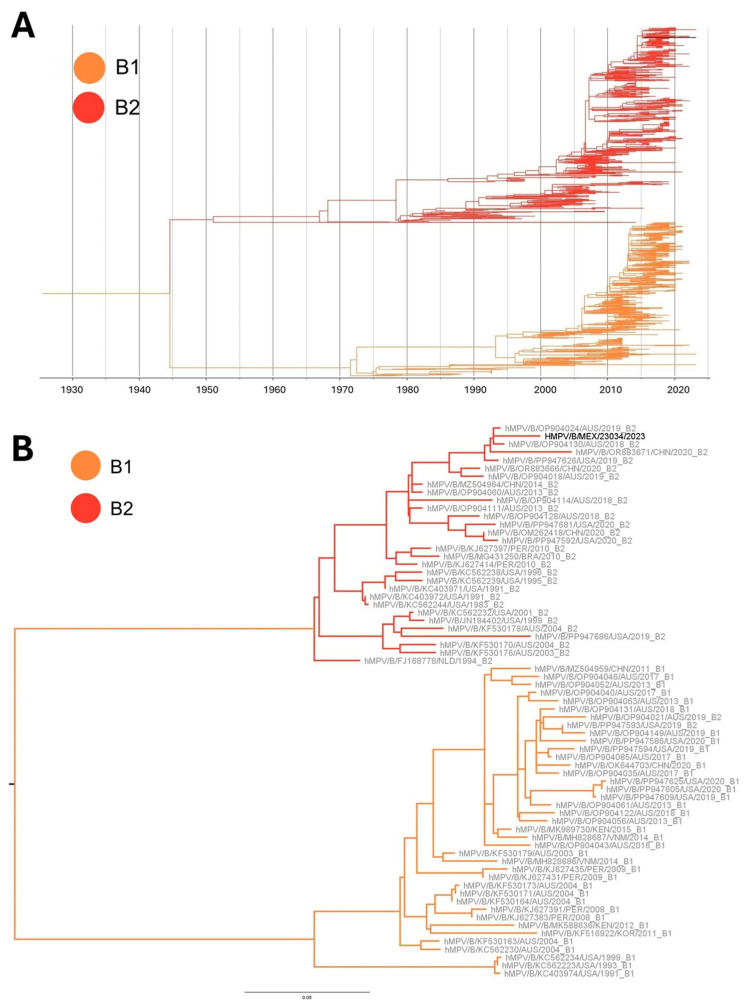
(**A**) Time-scaled maximum likelihood phylogenetic tree constructed using all the available G gene sequences of human metapneumovirus B (hMPV-B). Branches are colored according to their lineage assignment (B1, B2), while the black branch represents the single hMPV-B sequence obtained in this study. (**B**) Maximum likelihood phylogenetic tree including the hMPV-B sequence obtained in this study (black-labeled taxon), alongside representative reference sequences for both B1 and B2 lineages (gray-labeled taxa).

The HMPV-A phylogenetic tree did not show clustering of SLP sequences as a single monophyletic cluster (Figure 1 and Figure 2). To the contrary, they were associated with different groups closely related to strains from the United States (2024), Czech Republic (2024), or Spain (2021). Samples HMPV/A/MEX/23008/2023, HMPV/A/MEX/23010/2023, and HMPV/A/MEX/23012/2023 did not group with other SLP sequences and were closely related to the NSVH2021-42-51282 (Spain, 2021; GenBank accession: OM243713.1), HMPV/NL/4/20/A (Netherlands, 2020; GenBank accession: ON461530.1), and HMPV/IND/JIPMER/RES-179/2023 (India, 2023; GenBank accession: PP502417.1) strains, respectively. As for the HMPV-B sequence, the phylogenetic analysis showed a close relation to strain NSVH2019-52-58648 (Spain, 2019; GenBank accession: OM243531.1). The relationship between the sequences detected in Mexico and sequences reported from other countries is shown in Supplementary Figure S1.

### 3.5. Time-Scaled Phylogenetic Analysis

Time-scaled ML phylogenies inferred with TreeTime showed that the HMPV-A sequences obtained in this study were distributed across distinct branches interspersed with strains collected in different geographic regions (United States, Czech Republic, Spain, and India) between 2021 and 2024. The lack of a single monophyletic cluster comprising the local sequences, combined with their temporal dispersion, supports the occurrence of multiple independent introduction events rather than a sole introduction followed by local diversification.

The mean evolutionary rate estimated for the G gene sequences of HMPV was 4.2 × 10^−3^ substitutions/site/year for HMPV-A, and 5.4 × 10^−3^ substitutions/site/year for HMPV-B, consistent with previously reported values for this gene, reinforcing the reliability of the phylogenetic calibration and supporting the interpretation that the observed genetic diversity in local strains reflects repeated introductions of divergent viral lineages circulating globally. The corresponding time-scaled phylogenetic tree, with zoomed views of the clades containing local sequences, is provided as Supplementary Figure S1.

### 3.6. Lineages Assignment

Among the 34 sequences obtained in the present study, 32 (94.1%) were assigned to the A2b2 genotype, one was assigned to the A2b1 genotype, and one to the B2 genotype (Figure 1 and Figure 2). Notably, all but one of the A2b2 sequences carried the 111-nt duplication in the hypervariable region, while the remaining sequence carried the 180-nt duplication. The sample assigned to the A2b1 genotype did not have a duplication.

The majority (1821/1840; 98.9%) of the HMPV-A sequences from the GenBank dataset were successfully assigned to a specific lineage: A1 (123, 6.7%), A2a (127, 7.2%), A2b1 (337, 18.3%), and A2b2 (1234, 67.8%). On the other hand, 1580/1581 (99.9%) of the HMPV-B sequences were assigned to the B1 (695, 44%) and B2 (885, 56%) genotypes.

The demographic and clinical characteristics of children in whom genotyping was carried out is shown in Table 1. No statistical analysis was carried out to compare the characteristics of children with infections caused by the different genotypes, since we identified only one infection caused by HMPV genotype A2b1 and one caused by HMPV genotype B2.

### 3.7. Predicted Glycosylation Sites

**Figure 3:**
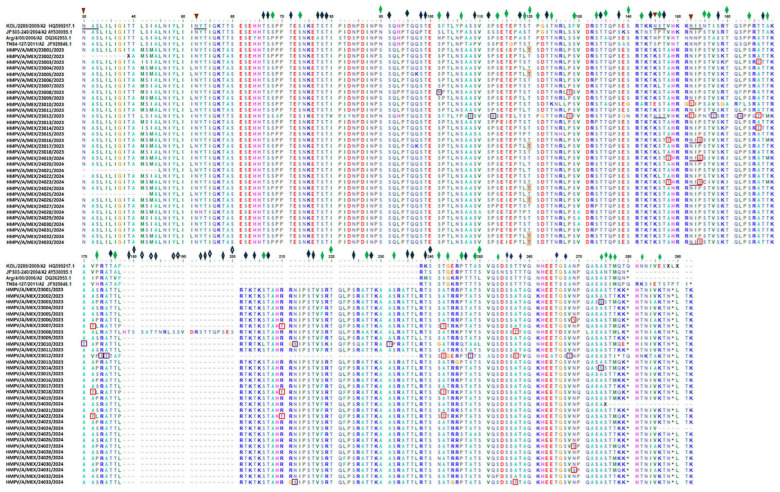
N and O-linked glycosylation sites predicted in SLP HMPV-A G protein deduced aminoacid sequences. (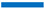) Sequons for N-glycosylation sites describes by Agrawal et al. [31] (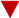) N-glycosylation sites predicted in in HMPV-A G protein reported by Agrawal et al. [31] (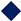) Predicted O-glycosylation sites conserved in all the SLP sequences. (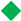) Predicted O-glycosylation sites not conserved in the SLP sequences. (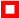) Predicted O-glycosylation sites unique in a sequence. (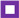) Predicted O-glycosylation sites that appear in at most four SLP sequences. (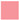) Threonine Residues predicted as not O-glycosylated.

**Figure 4:**
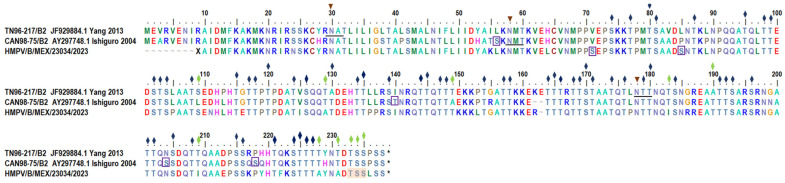
N and O-linked glycosylation sites predicted in SLP HMPV-B G protein deduced aminoacid sequence. (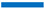) Sequons for N-glycosylation sites describes by Ishiguro et al. [32] and Yang et al. [33] (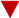) Possible N-glycosylated sites reported by Ishiguro et al. [32] and Yang et al. [33] and predicted in SLP sequence. (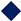) Predicted O-glycosylation sites conserved in the three showed sequences. (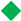) Predicted O-glycosylation sites not conserved in the three showed sequences. (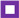) Predicted O-glycosylation sites unique in a sequence. (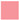) Residues predicted as not O-glycosylated.

Glycosylation sites for HMPV-A sequences were assessed using the amino acid sequence of the G glycoprotein from isolate KOL/2289/2009 (GenBank accession: HQ599217.1) (Figure 3). In all 29 HMPV-A sequences in which the transmembrane domain sequence was available, an N-linked glycosylation site (NGS), NAS sequon, was predicted at aa 30 (*p* > 0.75). In addition, in 33/33 of the HMPV-A extracellular domain sequences, an NGS was predicted in aa 52 (*p* > 0.75). At this position, one sequence showed a different sequon (NHT) compared to the reference sequences but conserved the predicted site; the rest show the same conserved sequence pattern (NYT sequon). An additional NGS (NIS sequon) was predicted in 4/33 of the sequences in the ectodomain (aa 152) (*p* > 0.5). The sample HMPV/A/MEX/23012/2023 was unique, with a predicted site at position 145 (NST sequon) (*p* > 0.5). Glycosylation site positions for the HMPV-B sequence were assessed using the deduced amino acid sequence of the G glycoprotein from isolate TN96-217 (GenBank accession: JF929884.1) as the reference (Figure 4). An NGS (NAT sequon) at aa 30 was predicted (*p* > 0.5). Two additional NGSs were predicted in the ectodomain sequence at position 58 (NMT) and position 178 (NTT) (*p* > 0.5).

The number of predicted O-linked glycosylation sites (OGS) in the HMPV-A sequences ranged from 61 (HMPV/A/MEX/23010/2023; 0.567737 < *p* < 0.988769) to 77 (HMPV/A/MEX/24021/2024; 0.506149 < *p* < 0.97268). Conservation of several predicted OGSs was observed in all sequences. However, some sequences showed a few predicted OGSs that were not present in the remaining sequences (Figure 3). Furthermore, an OGS was predicted in a serine residue at position 154 belonging to a NIS sequon, in which the asparagine was also predicted as N-glycosylated in four samples (HMPV/A/MEX/23016/2023, HMPV/A/MEX/24019/2024, HMPV/A/MEX/24023/2024, and HMPV/A/MEX/24033/2024) (Figure 3). A similar prediction also occurred at position 147 in sample HMPV/A/MEX/23012/2023. In this case, the threonine in the NST sequon was predicted as an OGS. In addition, six sequences were predicted as not O-glycosylated at position 120, despite the presence of a threonine residue. The phylogenetic inference analysis for the HMPV-A G gene sequences revealed that 5/6 of these sequences grouped into the same branch within the A2b2 lineage, also sharing the loss of a predicted OGS by a S68P aa change (Figure 3 and Supplementary Figure S2). In this line, in another cluster (A2b2 lineage) three sequences (HMPV/A/MEX/23007/2023, HMPV/A/MEX/23018/2023, and HMPV/A/MEX/24022/2024) share unique OGSs compared with the rest of the sequences at positions 171, 210, and 242. Furthermore, three additional sequences (HMPV/A/MEX/23006/2023, HMPV/A/MEX/24027/2024, and HMPV/A/MEX/24031/2024) grouped in a different cluster (A2b2 lineage) share a unique OGS at position 269 (Figure 3 and Supplementary Figure S2).

Another interesting observation was the cluster grouping 3/4 sequences (HMPV/A/MEX/23016/2023, HMPV/A/MEX/24019/2024, and HMPV/A/MEX/24023/2024) with predicted NGS at position 152 (NIS sequon). In contrast, the sequence of sample HMPV/A/MEX/23012/2023, in which nine unique OGSs were predicted (positions 108, 112, 152, 157, 162, 173, 174, 248, and 268), formed a single branch in a cluster of sequences from the A2b1 lineage.

The presence of some OGSs and the loss of NGSs was observed in multiple HMPV-A sequences within the 37 amino acid duplication region (encoded by the 111 nt duplication) in comparison to the original sequence in the isolate KOL/2289/2009 (Figure 3). An example is the aa position 232, where 14/32 sequences gained an OGS by the change of proline (position 172 in the original region) by serine. In contrast, 9/32 samples lost OGSs at aa 218 due to changes of serine (completely conserved at position 158 in the original region) by phenylalanine. Moreover, the NGSs and OGSs mentioned above in positions 152 and 154, respectively, in the original region of samples HMPV/A/MEX/23016/2023, HMPV/A/MEX/24019/2024, and HMPV/A/MEX/24023/2024 also disappeared in the duplicated region via an S214P change. In sample HMPV/A/MEX/24033/2024, two changes occurred in the duplication region, leading to the interchange of an NGS (aa 152 in the original non-duplicated region) with an OGS in position 212.

The number of predicted OGSs in the HMPV-B sequence was 61 (0.84218 < *p* < 0.998552), including two unique OGSs compared with all other sequences in the alignment as shown in Figure 4. In addition, two OGSs were predicted at positions 179 and 180 inside an NTT sequon. In contrast, no predicted OGSs were predicted at positions 233–235.

## 4. Discussion

In our study, we identified a predominance of HMPV-A strains during the 2023–2024 winter season in SLP. Notably, most sequences belonged to the A2b2 genotype but did not form a monophyletic group. A similar distribution was described by Goya et al. [34], suggesting ongoing virus introductions and the appearance of diverse variants within sublineages. Lee et al. [35] also reported different subclusters within the A2.2.2 (A2b2) branch of the G glycoprotein gene phylogeny from viruses detected in Taiwan. Parida et al. [36] described separate clusters in the HMPV A2c group (A2b2) in India. Altogether, the available data indicate that several sublineages within the A2b2 genotype circulate globally, and no regional clustering appears to be present. Such variability may have implications for viral tropism, pathogenesis, or immune escape.

Little is known regarding HMPV epidemiology and genotype distribution in Mexico. Previous studies have identified HMPV as an important pathogen. In a study carried out in SLP between 2002 and 2004, we identified HMPV in 6.1% of children under 3 years of age hospitalized with respiratory infections, and this virus was the second most common etiological agent in this population after HRSV [25]. A more recent study conducted in the state of Jalisco identified HMPV as the most frequent virus associated with acute respiratory infections in children under 5 years of age (22%) [26]. The prevalence of HMPV in the present work during the 2023–2024 season (17.4%) was close to this figure, underscoring the importance of this virus as a cause of severe infections in children. Of note, the study carried out in SLP between 2002 and 2004 is the only study which has previously analyzed the genotype distribution of HMPV in Mexico [25]. In that work, HMPV genotypes were determined in 26 samples based on the N gene sequence; twenty-three samples (88.5%) corresponded to the A2 genotype, and three samples (11.5%) were grouped in the B2 genotype. In the present study, we identified for the first time the circulation of the A2b1 and A2b2 HMPV sublineages in Mexico.

During the last decade, starting before the SARS-CoV-2 pandemic, a notable increase in the prevalence of A2b2 strains with 111-nt or 180-nt duplications present in the G glycoprotein gene occurred. This global trend has been reported by several researchers, and these viruses, particularly A2b2-111 nt-duplication, have displaced other HMPV-A viruses in Asia, Europe, South America, and very recently in North America [34,37,38]. The results of the phylogenetic analysis and the lineage assignment made in the present work are in accordance with the trend that has been observed since 94.1% of the SLP HMPV-A G gene sequences were assigned to the A2b2 genotype, and all of them carry a duplication region. An analysis of the HMPV G and F gene of strains from hospitalized Taiwanese children in 2023 revealed that more than 70% of HMPV-A sequences had the 111 nt-duplication [35]. Also, Piñana et al. [39] reported the prevalence of viruses with the 111 nt-duplication in HMPV-A samples from Spain in a period from 2020–2022. In that study, the authors described a drastic change from alternating shifts in predominance between HMPV-A and HMPV-B to a majority of viruses containing the 111-duplication. More recently, Goya et al. [34] reported the analysis of HMPV genomes collected from 2021 to 2025 (including 13 samples collected between November 2024 and January 2025) from symptomatic patients from all age groups in western Washington State in the United States. They observed co-circulation of viruses belonging to four sublineages: A2b1, A2b2-111 nt-duplication, B1, and B2. In addition, they described the A2b2 lineage as the most prevalent, and all of the lineages carried a 111-nt G gene duplication. Also in 2025, Fadeev et al. [40] described a whole-genome sequencing study of 44 HMPV strains circulating from 2017 to 2024 in various regions of the Russian Federation. HMPV-positive samples collected from patients of different ages with acute respiratory viral infections were analyzed. The authors found that most of the viruses belonged to the A2b2 lineage, and they detected the 111 nt G gene duplication in 84% of sequences.

Goya et al. [34] calculated the evolution rate of lineage A2b2-111-duplication as 1.1 × 10^−3^ substitutions per site per year and demonstrated that it is a fast-evolving lineage. Formerly, Piñana et al. described duplications in HMPV as a possible evolutionary advantage due to an “improved steric shielding” [39]. They predicted that G proteins with either duplication (111-nt or 180-nt) protrude more from the membrane than the wild type. A former suggestion of a shielding function of G toward the F protein to mask its antigenic epitopes was supported by their results [38,41]. Such an effect has also been reported for HRSV strains harboring partial duplications in the G gene [42].

Viral proteins are often highly glycosylated, especially those targeted by the host’s immune system. Glycosylation tends to be dynamic over time as viruses propagate in host populations, leading to increased numbers of glycosylation sites in response to the immune system and other pressures referred to in [43]. In the case of HMPV, the NGS number predicted in the G glycoproteins from different subgroups varied from two to seven. A conserved NGS (NAS sequon for HMPV-A and NAT for HMPV-B) at aa 30 has been previously reported [31,32,33]. These observations were corroborated in the present work, since these NGS were predicted in all of the HMPV-A transmembrane domain sequences as well as in the HMPV-B sequence.

Three conserved NGSs have been reported by other authors among A2 subgroup sequences at aa positions 52 (NYT), 145 (NST) and 152 (NIS) in the G glycoprotein extracellular domain [31,32,33]. Those NGSs were also predicted in sequences obtained in the present study (Figure 3). However, only the site in aa 52 was conserved in most sequences (32/33, 97%). In contrast, the predicted site at position 145 was present in only one sequence (HMPV/A/MEX/23012/2023). In addition, the predicted NGS in aa 152 was not present in 27/33 (81.8%) of the sequences (Figure 3). While some viruses without these NGS have been reported previously, these have been uncommon. Agrawal et al. [31] reported that only 1/17 A2 strains had lost the NGS at amino acid position 145 and 1/17 at position 152.

The NGSs predicted at amino acid 58 (NMT) and amino acid 178 (NTT) in the HMPV/B/MEX/23034/2023 (B2) sequence had been described as conserved in B2 subgroup by Ishiguro et al. [32] and Yang et al. [33].

In comparison with potential NGS, a larger number of potential OGS have been reported for HMPV strains. The number of potential acceptors of O-glycosylation reported by different authors varies: Agrawal et al. [31] identified 45 to 55 serine and threonine residues in the G glycoprotein extracellular region to be potentially O-glycosylated in subgroup A2 and B1 strains, while Galiano et al. [44] identified 49–72 sites among all HMPV strains. In addition, Yang et al. [44] reported a larger number of OGS, which varied according to HMPV subgroup (77–84 for A1, 67–78 for A2, 65–69 for B1, and 64–76 for B2). Overall, predicted OGS have been located after amino acid 66 in previous reports [33,41]. Similarly, sequences from SLP showed a large number of potential OGS, ranging from 61 to 77.

A notable feature displayed by HMPV sequences is the presence of sequons that carry both predicted NGS and OGS, such as NIS (at positions 145/147 of HMPV-A), NST (at positions 152/154 of HMPV-A), and NTT (at positions 178/180 of HMPV-B). The occurrence of both N- and O-glycosylations within an N-sequon has been previously described in human genes and in the SARS-CoV-2 S protein by Chandrasekhar et al. [45], Riethmueller et al. [46], and Sanda et al. [47]. Of interest, Tian et al. [48] carried out site-directed mutagenesis experiments and found that glycosylated asparagine is a requirement for O-glycosylation related to N-sequon. Based on these results, the authors proposed an “O-Follow-N” rule, where O-glycosylation occurs near the glycosylated asparagine in an N-sequon. However, they did not study the relevance of this rule for S glycoprotein.

This work is not without limitations. One of them is that the complete nucleotide and amino acid sequences of the G protein could not be obtained for analysis. Another aspect considered as a limitation is that glycosylation analyses are based on predictions; the existence of the predicted glycosylation sites requires experimental confirmation with the use of modern tools. However, despite these limitations, the findings of this research are still relevant.

## 5. Conclusions

The detailed description of HMPV genotypes and the diverse arrays of G protein N- and O-linked glycosylation patterns present in a Mexican pediatric population during the post-pandemic period, described for the first time in this work, contributes to the understanding of global spread and evolution of HMPV strains, especially those classified as A2b2 genotypes with a 111 nt duplication. In addition, the present research provides a baseline reference for future HMPV molecular epidemiology surveillance in SLP and Mexico.

## Figures and Tables

**Table 1:** Demographic and clinical characteristics of children with HMPV infection in whom the viral genotype was determined.

Characteristics		Infants with HMPV Genotype (n = 34)	A2b2 Genotype (n = 32)	A2b1 Genotype (n = 1)	B2 Genotype (n = 1)
Sex	Female	13 (38.2%)	12 (37.5%)	0	1
	Male	21 (61.8%)	20 (62.5%)	1	0
Age	0-<12 months	15 (44.1%)	15 (46.9%)	0	0
	12-<24 months	10 (29.4%)	8 (25%)	1	1
	24-<36 months	9 (26.4%)	9 (28.1%)	0	0
Underlying conditions					
Congenital heart disease		2 (5.9%)	2 (6.25%)	0	0
Bronchopulmonary dysplasia		1 (2.9%)	1 (3.12%)	0	0
Down syndrome		3 (8.8%)	3 (9.4%)	0	0
Immunodeficiency		1 (2.9%)	1 (3.12%)	0	0
Asthma		6 (17.6%)	6 (18.75%)	0	0
Preterm birth		4 (11.8%)	4 (12.5%)	0	0
Breastfeeding history		29 (85.3%)	27 (84.4%)	1	1
Tobacco smoke exposure		8 (23.5%)	7 (21.9%)	1	0
Wood smoke exposure		3 (8.8%)	3 (9.4%)	0	0
Siblings < 5 years old		11 (32.3%)	10	0	1
Day-care attendance		1 (2.9%)	1 (3.12%)	0	0
Signs and symptoms					
Cough		34 (100%)	32 (100%)	1	1
Respiratory distress		34 (100%)	32 (100%)	1	1
Fever		29 (85.3%)	27 (84.4%)	1	1
Crackles		29 (85.3%)	27 (84.4%)	1	1
Rhinorrhea		30 (88.2%)	28 (87.5%)	1	1
Wheezing		14 (41.2%)	14 (43.75%)	0	0
Intensive care unit admission		2 (5.9%)	2 (6.25%)	0	0
Death		1 (2.9%)	1 (3.12%)	0	0

## Data Availability

Data used for this study are available at public repositories including GenBank and GISAID. Local sequences reported in this study were submitted to GenBank and the assigned accession numbers are PV950564-PV950597.

## Supplementary Materials

The following supporting information can be downloaded at: https://www.mdpi.com/article/10.3390/v17101338/s1, Supplementary Figure S1. Time-scaled maximum likelihood phylogenetic tree of the HMPV G gene sequences. Local sequences from San Luis Potosí (shown in black) are distributed across different clades and intermixed with strains from diverse geographic regions (United States, Czech Republic, Spain, and India). Zoomed views highlight the placement of San Luis Potosí sequences, illustrating that they do not form a single monophyletic cluster but rather group with viruses from distinct locations, supporting the interpretation of multiple independent introductions; Figure S2. Common or unique amino acid changes causing the loss or gain of N- and O-linked glycosylation sites in SLP HMPV-A G glycoprotein sequences grouped by gene phylogenetic analysis; Supplementary Table S1. Set of primers used for detection or genotyping of HMPV in samples from SLP-México [16,20,28], August 2023–August 2024; Supplementary Table S2. Sequences used as Genotype Reference obtained from Nextstrain; Supplementary Table S3. Demographic and clinical characteristics of children with HMPV infection in whom the viral genotype was obtained and those in which it was not determined.

## Abbreviations

The following abbreviations are used in this manuscript:

HMPV	Human Metapneumovirus
SLP	San Luis Potosí
LRTI	Lower respiratory tract infections
nt	nucleotide
Ct	Cycle threshold
NGS	N-linked glycosylation site
OGS	O-linked glycosylation site
aa	amino acid
HRSV	Human Respiratory Syncytial Virus
