# Supplementary material for: Molecular Epidemiology of Human Metapneumovirus Infections in Children from San Luis Potosí-Mexico
Source: Viruses. 2025 Oct 2;17(10):1338. doi: 10.3390/v17101338 (PMC12567928; doi:10.3390/v17101338)
Supplement: Supplementary file 1 [file viruses-17-01338-s001.zip › viruses-3864423-supplementary.pdf]

**Molecular Epidemiology of Human Metapneumovirus Infections in Children from San Luis Potosí-Mexico**

Nadia Martínez-Marrero, Juan Carlos Muñoz-Escalante, Jan Michell Yerena-Rivera, Luis R. Jaime-Rocha, José J. Leija-Martínez, Ana M. González-Ortiz, Daniel E. Noyola

## Supplementary Tables And Figure

**Table S1.** Set of primers used for detection or genotyping of HMPV in samples from SLP-México, August 2023-August 2024.

| Primer name | Sequence (5´-3´) | Gene | Anneling temperature (ºC) | Product size (bp) | Reference |
|---|---|---|---|---|---|
| **NIIDN1-F** | TGATGCRCTCAAAAGATACCC | N | 58 | 159 | Sugimoto et al. [28] |
| **NIID-R** | GCAAAGCAGAAAGTTTRTTYGT | | | | Adapted from Sugimoto et al. [28] |
| **SH7A** | TAAAAACAAAAATATGGGACAAG | G | 62.8 | 1062 | Adapted from van der Hoogen et al. [16] |
| **GNR2A** | GGATTCATTAAGAGGATCCATTG | | | | Saikusa et al. [20] |
| **SH7B** | TAYAAAACAAGAACATGGGACAAG | G | 55 | 979 | Adapted from van der Hoogen et al. [16] |
| **GNR2B** | RAGATAAACATTRACAGTRGAYTC | | | | Adapted from Saikusa et al. [20] |

**Table S2.** Sequences used as Genotype Reference obtained from Nextstrain.

| Accession Number | Genotype | Accession Number | Genotype | Accession Number | Genotype | Accession Number | Genotype |
|---|---|---|---|---|---|---|---|
| KC403980 | A1 | KC403978 | A2b1 | KC403974 | B1 | OP904131 | B1 |
| KC562226 | A1 | KJ627381 | A2b1 | KC562223 | B1 | OP904149 | B1 |
| KC562236 | A1 | KJ627396 | A2b1 | KC562234 | B1 | PP947593 | B1 |

| | | | | | | | |
|---|---|---|---|---|---|---|---|
| KU821121 | A1 | KJ627411 | A2b1 | KC562230 | B1 | OP904021 | B1 |
| JN184400 | A2a | KJ627437 | A2b1 | KF530163 | B1 | KC562244 | B2 |
| KC403979 | A2a | KJ627406 | A2b1 | KF516922 | B1 | KF530176 | B2 |
| KC403982 | A2a | KJ627384 | A2b1 | MK588636 | B1 | KF530170 | B2 |
| KC403981 | A2a | KJ627377 | A2b1 | KJ627383 | B1 | PP947686 | B2 |
| KJ627398 | A2a | KJ627387 | A2b1 | KJ627391 | B1 | KF530178 | B2 |
| KJ627390 | A2a | KJ627399 | A2b1 | KF530164 | B1 | JN184402 | B2 |
| KJ627427 | A2a | KJ627429 | A2b1 | KF530171 | B1 | KC562232 | B2 |
| KJ627416 | A2a | KJ627426 | A2b1 | KF530173 | B1 | FJ168778 | B2 |
| KJ627425 | A2a | KJ627386 | A2b1 | KJ627431 | B1 | KC403972 | B2 |
| KJ627378 | A2a | KJ627424 | A2b1 | KJ627435 | B1 | KC403971 | B2 |
| KJ627419 | A2a | KJ627404 | A2b1 | MH828686 | B1 | KC562239 | B2 |
| KJ627395 | A2a | KJ627403 | A2b1 | KF530179 | B1 | KC562238 | B2 |
| KJ627433 | A2a | KJ627410 | A2b1 | MZ504959 | B1 | KJ627414 | B2 |
| KJ627413 | A2a | GQ15365 | A2b2 | MH828687 | B1 | MG431250 | B2 |
| KC56222 | A2b1 | MW22198 | A2b2 | MK989730 | B1 | KJ627397 | B2 |
| KC56223 | A2b1 | MN86746 | A2b2 | OP904043 | B1 | MG431250 | B2 |
| KJ627392 | A2b1 | MZ504958 | A2b2 | OP904046 | B1 | KJ627397 | B2 |
| KJ627382 | A2b1 | OP904059 | A2b2 | OP904046 | B1 | OP904060 | B2 |
| KJ627401 | A2b1 | MK588634 | A2b2 | OP904052 | B1 | MZ504964 | B2 |
| KJ627385 | A2b1 | OP904039 | A2b2 | OP904056 | B1 | PP947592 | B2 |
| KJ627402 | A2b1 | PP947649 | A2b2 | OP904061 | B1 | OM262418 | B2 |
| KJ627417 | A2b1 | LC671558 | A2b2 | OP904122 | B1 | PP947681 | B2 |
| KJ627379 | A2b1 | PP315925 | A2b2 | PP947609 | B1 | OP904128 | B2 |
| KJ627405 | A2b1 | MK087726 | A2b2 | PP947605 | B1 | OP904111 | B2 |
| KJ627430 | A2b1 | PP947599 | A2b2 | PP947625 | B1 | OP904114 | B2 |
| KC56224 | A2b1 | PP947699 | A2b2 | OP904063 | B1 | OP904018 | B2 |
| MK98973 | A2b1 | PP947643 | A2b2 | OP904040 | B1 | OR883666 | B2 |
| MK98973 | A2b1 | OZ211385 | A2b2 | OP904035 | B1 | PP947626 | B2 |
| KC56222 | A2b1 | OZ211642 | A2b2 | OK644703 | B1 | OP904130 | B2 |
| KF530190 | A2b1 | | | OP904085 | B1 | OP904024 | B2 |
| KC40398 | A2b1 | | | PP947594 | B1 | OR883671 | B2 |
| KC40398 | A2b1 | | | PP947586 | B1 | | |

**Table S3.** Demographic and clinical characteristics of children with HMPV infection in whom the viral genotype was obtained and those in which it was not determined

| Characteristics | No genotype (n=34) | Genotyped (n=34) | P |
|---|---|---|---|
| Sex Female | 15 (44.1%) | 13 (38.2%) | 0.62 |
| Male | 19 (55.9%) | 21 (61.8%) | |

| | | | |
|---|---|---|---|
| Age 0-<12 months<br>12-<24 months<br>24-<36 months | 20 (58.8%)<br>6 (17.6%)<br>8 (23.5%) | 15 (44.1%)<br>10 (29.4%)<br>9 (26.4%) | 0.41 |
| | | | |
| Underlying conditions | | | |
| Congenital heart disease | 0 (0%) | 2 (5.9%) | 0.49 |
| Bronchopulmonary dysplasia | 1 (2.9%) | 1 (2.9%) | 0.99 |
| Down syndrome | 1 (2.9%) | 3 (8.8%) | 0.61 |
| Immunodeficiency | 0 (0%) | 1 (2.9%) | 0.99 |
| Asthma | 1 (2.9%) | 6 (17.6%) | 0.105 |
| Preterm birth | 3 (8.8%) | 4 (11.8%) | 0.99 |
| | | | |
| Breastfeeding history | 26 (76.5%) | 29 (85.3%) | 0.35 |
| Tobacco smoke exposure | 13 (38.2%) | 8 (23.5%) | 0.19 |
| Wood smoke exposure | 2 (5.9%) | 3 (8.8%) | 0.99 |
| Siblings <5 years old | 15 (44.1%) | 11 (32.3%) | 0.32 |
| Day-care attendance | 3 (8.8%) | 1 (2.9%) | 0.61 |
| | | | |
| Cough | 34 (100%) | 34 (100%) | NA |
| Respiratory distress | 34 (100%) | 34 (100%) | NA |
| Fever | 28 (82.3%) | 29 (85.3%) | 0.74 |
| Crackles | 28 (82.3%) | 29 (85.3%) | 0.74 |
| Rhinorrhea | 27 (79.4%) | 30 (88.2%) | 0.32 |
| Wheezing | 9 (26.5%) | 14 (41.2%) | 0.2 |
| | | | |
| Intensive care unit admission | 2 (5.9%) | 2 (5.9%) | 0.99 |
| Death | 1 (2.9%) | 1 (2.9%) | 0.99 |

**Supplementary Figure S1**

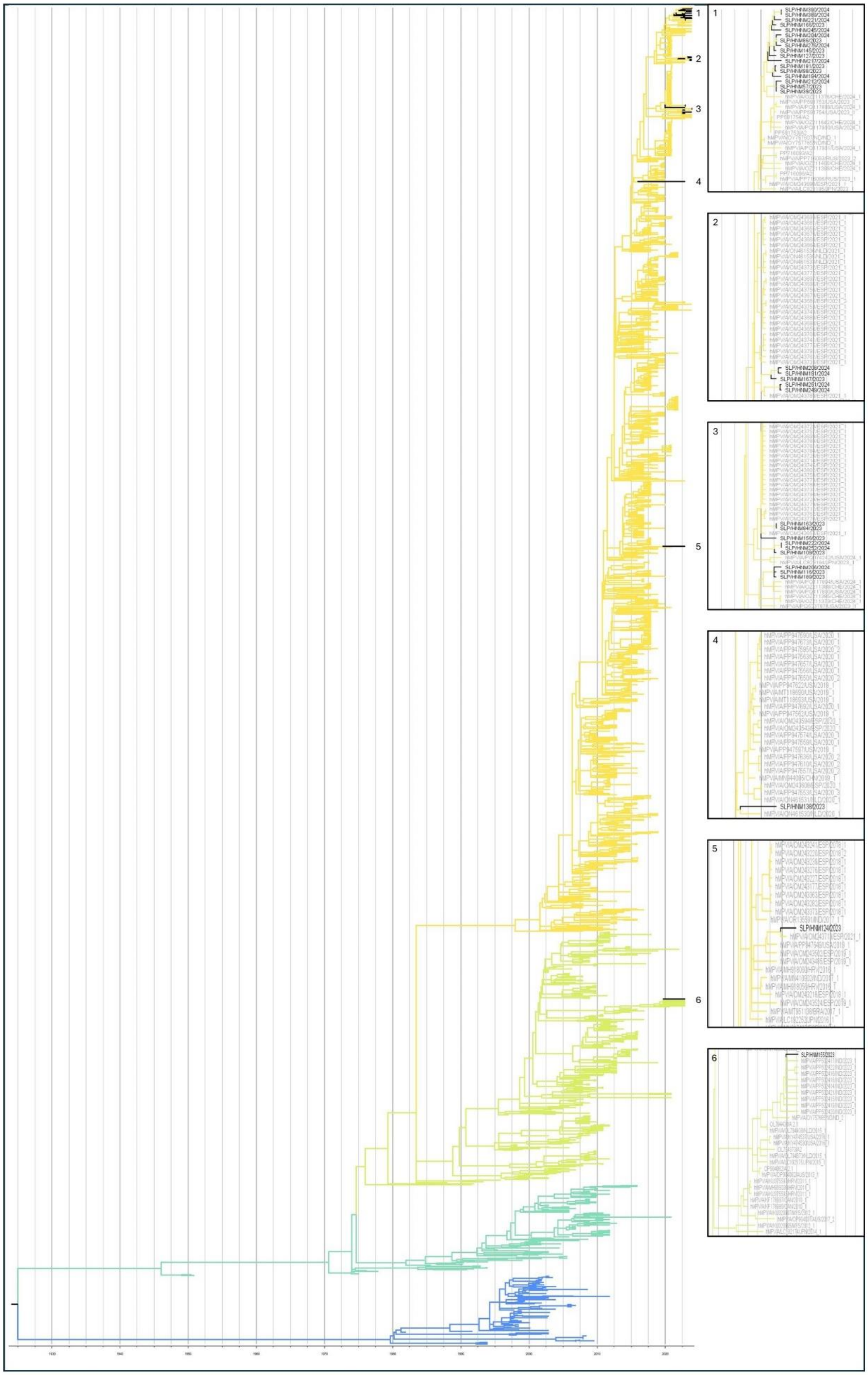

**Figure S1.** Time-scaled maximum likelihood phylogenetic tree of the HMPV G gene sequences. Local sequences from San Luis Potosí (shown in black) are distributed across different clades and intermixed with strains from diverse geographic regions (United States, Czech Republic, Spain, and India). Zoomed views highlight the placement of San Luis Potosí sequences, illustrating that they do not form a single monophyletic cluster but rather group with viruses from distinct locations, supporting the interpretation of multiple independent introductions.

## Supplementary Figure S2

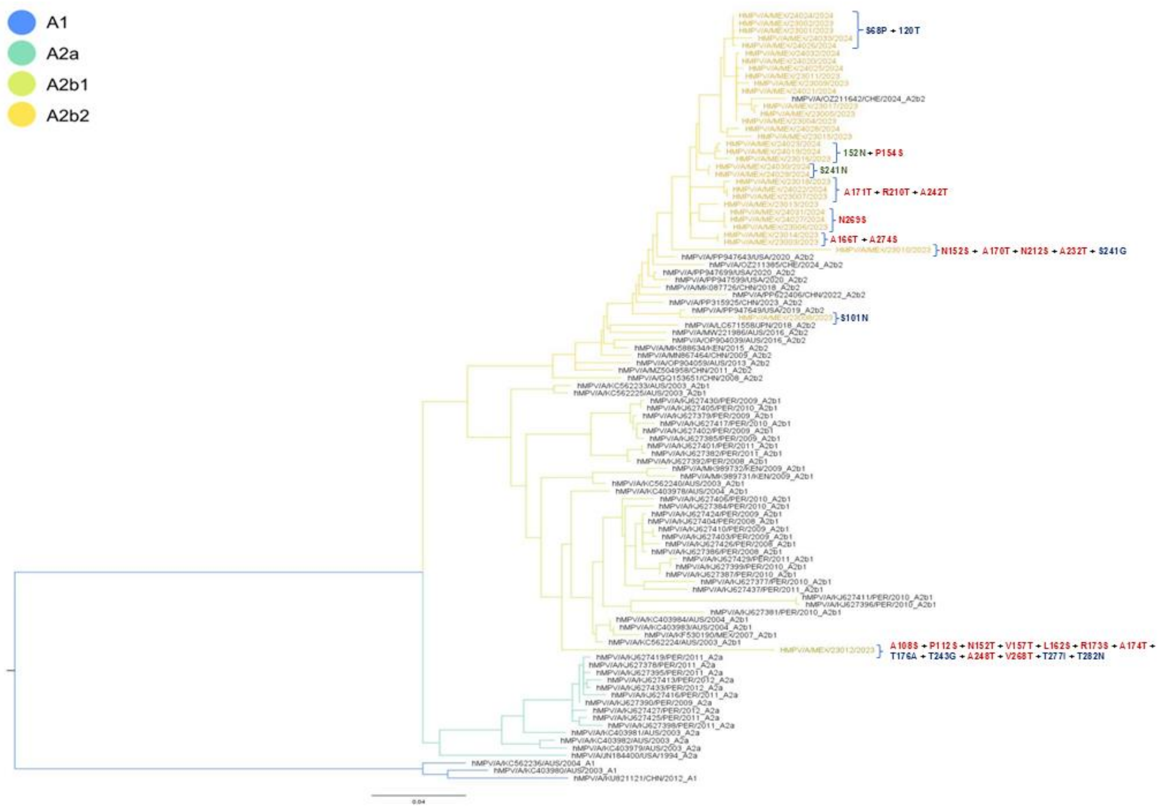


**Figure S2.** Common or unique amino acid changes causing the loss or gain of N- and O-linked glycosylation sites in SLP HMPV-A G glycoprotein sequences grouped by gene phylogenetic analysis. (Red color) Gain of a predicted O-glycosylation site. (Blue color) Loss of a predicted O-glycosylation site. (Green color) Gain or shared predicted N-glycosylated sites. Sequences were compared with isolate KOL/2289/2009 and between them.

## References

16. van den Hoogen, B.G.; Herfst, S.; Sprong, L.; Cane, P.A.; Forleo-Neto, E.; de Swart, R.L.; Osterhaus, A.D.; Fouchier, R.A. An-tigenic and genetic variability of human metapneumoviruses. *Emerg. Infect. Dis*. **2004**, *10*, 658–666

20. Saikusa, M.; Nao, N.; Kawakami, C.; Usuku, S.; Sasao, T.; Toyozawa, T.; Takeda, M.; Okubo, I. A novel 111-nucleotide duplication in the G gene of human metapneumovirus. *Microbiol. Immunol*. **2017**, *61*, 507–512.

28. Sugimoto, S.; Kawase, M.; Suwa, R.; Kakizaki, M.; Kume, Y.; Chishiki, M.; Ono, T.; Okabe, H.; Norito, S.; Hosoya, M.; et al. Development of a duplex real-time RT-PCR assay for the detection and identification of two subgroups of human metapneumovirus in a single tube. *J. Virol. Methods* **2023**, *322*, 114812.
